# Impact of chronic kidney disease on the short- and long-term outcomes of laparoscopic gastrectomy for gastric cancer patients

**DOI:** 10.1371/journal.pone.0250997

**Published:** 2021-04-30

**Authors:** Katsunobu Sakurai, Naoshi Kubo, Yutaka Tamamori, Naoki Aomatsu, Takafumi Nishii, Akiko Tachimori, Kiyoshi Maeda

**Affiliations:** Department of Gastroenterological Surgery, Osaka City General Hospital, Osaka, Japan; University of Bari: Universita degli Studi di Bari Aldo Moro, ITALY

## Abstract

**Backgrounds:**

This study was undertaken to investigate the impact of coexisting chronic kidney disease (CKD) on short- and long-term outcomes of laparoscopic gastrectomy in patients with gastric cancer (GC).

**Methods:**

We reviewed the data of 798 patients treated for GC by laparoscopic gastrectomy. All procedures took place between January 2010 and December 2017. Patients were divided into three groups according to their estimated glomerular filtration rate (eGFR): severe CKD group, 44 patients with eGFR < 45 mL/min/1.73 m^2^; moderate CKD group, 117 patients with 45 ≤ eGFR < 60; control group, 637 patients with eGFR ≥ 60.

**Results:**

Based on multivariate analysis, severe CKD (eGFR < 45) emerged as an independent predictor of anastomotic leak (Hazard ratio 4.63, 95% confidence interval [CI] 1.62–11.54). The 5-year overall survival (OS) rates by group were 46.3% (severe CKD), 76.6% (moderate CKD), and 81.5% (control). Multivariate analysis likewise identified severe CKD (eGFR < 45) as an independent correlate of poor 5-year OS. The 5-year cancer-specific survival (CSS) rates did not differ significantly by group.

**Conclusions:**

An eGFR value less than 45 mL/min/1.73 m^2^ is a useful factor for predicting both anastomotic leak and 5-year OS in GC patients undergoing laparoscopic gastrectomy. Clinical care to improve eGFR should be reinforced before and after gastrectomy for GC patients with severe CKD.

## Introduction

In East Asia, laparoscopic gastrectomy for gastric cancer (GC) is as safe and curative as open gastrectomy and has become the standard procedure for early GC [1, 2]. This procedure has been widely adopted for advanced cancer in recent years, with non-inferior outcomes compared to open gastrectomy [3, 4]. In addition, there have been several reports demonstrating that laparoscopic gastrectomy can be safely performed even in the elderly due to its minimally invasive nature [5, 6]. Thus, laparoscopic gastrectomy for GC is now an essential procedure for surgeons.

Japan has the highest aging rate worldwide, and the elderly population will continue to increase going forward [7, 8]. The elderly often have comorbidities, and the risk of operation and long-term outcomes differ depending on the severity of the comorbidities. Therefore, elucidating the association between the comorbidities and treatment outcomes for patients undergoing laparoscopic gastrectomy will help identify differences in surgical risk among individual patients and determine the appropriate treatment.

In the course of aging, renal function slowly declines in the absence of obvious symptoms. Thus, there are many cancer patients with untreated chronic kidney disease (CKD). As stipulated in various professional guidelines [9–11], classification of CKD currently relies on estimated glomerular filtration rates (eGFRs) as follows: G1, normal or high (eGFR ≥ 90 mL/min/1.73 m^2^); G2, mildly decreased (eGFR 60–89 mL/min/1.73 m^2^); G3a, mildly to moderately decreased (eGFR 45–59 mL/min/1.73 m^2^); G3b, moderately to severely decreased (eGFR 30–44 mL/min/1.73 m^2^); G4, severely decreased (eGFR 15–29 mL/min/1.73 m^2^); G5, renal failure (eGFR < 15 mL/min/1.73 m^2^, dialysis indicated).

The impact of CKD on gastrectomy for GC patients is controversial. Some reports have found that the incidence of morbidity correlated with CKD [12, 13]; however, another report found no significant relationship between short- and long-term outcomes and CKD [14]. Moreover, despite the prevalence of laparoscopic gastrectomy for GC, most patients enrolled in these studies underwent open gastrectomy. Thus, it is essential to elucidate the impact of CKD on treatment outcomes, specifically for patients undergoing laparoscopic gastrectomy. In this retrospective study, we investigated the treatment outcomes of such patients, focusing on coexisting CKD and using eGFR as a gauge of renal function.

## Methods

### Patients and clinical data collection

A total of 798 patients, who underwent laparoscopic gastrectomy for GC at Osaka City General Hospital (Osaka, Japan) between January 2010 and December 2017, were included in this study. Any patients subjected to prior gastrectomy were excluded. Clinicopathologic variables and postoperative complications were extracted from medical records, operative records, and pathology reports. For each patient, retrieval of baseline characteristics included age, gender, body mass index (BMI), serum albumin, comorbidities (diabetes mellitus or ischemic heart disease), tumor data (TNM stage), type of gastrectomy, operative time, recorded blood loss, harvested lymph node count, postoperative complications, and duration of postoperative hospital stay. Informed consent was obtained in the form of opt-out on the hospital’s web-site because the analysis used anonymous clinical data that were obtained after each patient agreed to treatment by written consent. The opt-out is a method of publishing information on the web without directly obtaining patient consent for clinical research, and ensuring that patients have the opportunity to refuse. We accessed patient’s medical records from January 2018 to January 2021. All data were fully anonymized before we analyzed them. This study was reviewed and approved by the Institutional Review Board (IRB) of Osaka City General Hospital (IRB2006051). The IRB of our hospital approved the use of an opt-out consent mechanism in this study.

### Definition

Comorbidity was classified as reported previously [15]. Ischemic heart disease was presumed in patients diagnosed with angina or myocardial infarction or having undergone stent placement, bypass surgery, or medical therapy for coronary disease. Diabetes mellitus was defined as use of oral hypoglycemic drugs or insulin or hemoglobin A1c (HbA1c) level ≧ 6.2, reflecting our institutional criteria. All pathologic terms and classifications were those authorized by the Japanese Gastric Association (14^th^ edition) [16]. Postoperative complications were graded according to Clavien-Dindo (CD) classification [17]. Leakage, pancreatic fistula, intraabdominal abscess and pneumonia of CD grade 2 and beyond were considered customary complications, whereas events of CD grade 3 or more constituted major complications. Overall survival (OS) is defined as the time from the operation date until the date of death. Cancer-specific survival (CSS) refers to the time from the operation until death from tumor relapse only.

### Assessment of renal function

We used preoperative eGFR values to assess renal function, based on preoperative blood test data recorded following admission. In accord with previous reports [10], the formulas used to calculate eGFR were: eGFR (mL/min/1.73 m^2^) = 194 × (serum creatinine)^-1.094^ × age^-0.287^ for males; eGFR = (194 × [serum creatinine]^–1.094^ × age^-0.287^) × 0.739 for females. In this study, patients were divided into three groups based on the CKD classification as reported previously [11, 12]: G1,2 (control group, eGFR ≥ 60); G3a (moderate CKD group, 45 ≤ eGFR < 60); G3b, 4, and 5 (severe CKD group, eGFR < 45).

### Laparoscopic procedures

We began performing laparoscopic gastrectomy at our facility in 1998 and used this procedure mainly for early GC [18, 19]. Since January 2010, we have adopted a laparoscopic approach for advanced GC. The reconstruction of the distal gastrectomy (DG) included the Billroth-II method with Brown’s anastomosis from January 2010 to March 2016. The gastrojejunostomy (GJ) was performed with an extracorporeal procedure from a small incision using a linear stapler from January 2010 to March 2013 and an intracorporeal procedure from April 2013 to March 2016. After April 2016, Billroth-I and II and Roux-en-Y (RY) reconstructions were performed with an intracorporeal procedure using a linear stapler in all parts. The reconstruction of a total gastrectomy (TG) was with an RY reconstruction in all cases. From January 2010 to March 2016, an esophagojejunostomy (EJ) was performed with an extracorporeal procedure from a small incision using a circular stapler. From April 2016, EJ was performed with an intracorporeal procedure using a linear stapler. The reconstruction of the proximal gastrectomy (PG) was double-tract from January 2010 to March 2014. Since April 2014, the double-tract or esophagogastrostomy (EG) was performed as a reconstruction method. EJ was performed with an extracorporeal procedure using a circular stapler from January 2010 to March 2016 and an intracorporeal procedure using a linear stapler since April 2016. EG was performed with an intracorporeal procedure using a linear stapler [20]. Surgeons qualified in the endoscopic surgical skill qualification system in Japan participated in all operations as either an operator or a first assistant.

### Statistical analysis

All statistical analyses were performed using standard software (JMP v11; SAS Institute Inc, Cary, NC, USA). Categorical variables were expressed as numerical values and percentages, and group data were compared via χ^2^ test or Fisher’s exact test. Continuous variables were expressed as median (minimum-maximum), and differences between the two groups (the control vs. moderate CKD group and the control vs. severe CKD group) were analyzed using Mann-Whitney U test. The uni- and multivariate analysis was performed using a logistic regression model to investigate the factors associated with the incidence of anastomotic leak. The univariate analysis included age, gender, BMI, albumin, co-morbidities (ischemic heart disease, diabetes mellitus and CKD), gastrectomy type, pStage, blood loss and operative time as covariates. All variables with a *p* value < 0.10 in the univariate analysis were entered into a multivariate analysis. Survival curves were generated by Kaplan-Meier method, analyzing differences by log-rank test. Univariate and multivariate hazard ratios (HRs) were calculated via Cox proportional hazard model. Variables that were found to be at *p* < 0.10 on univariate analysis were included in the multivariate analysis. All reported *p*-values were two-sided, setting statistical significance at *p* < 0.05.

## Results

### Characteristics of patients in each eGFR groups

Clinicopathologic and perioperative characteristics of the GC patients selected for study are shown in Table 1. There were 637 patients in the control group, 117 patients in the moderate CKD group and 44 patients in the severe CKD group. Mean patient age was significantly higher in the moderate and severe CKD group (vs. control) group (*p* < 0.001 for each), but gender ratios and BMI values did not differ significantly by group. In the severe and moderate CKD groups, the serum albumin levels were significantly lower than in the control group (*p* < 0.001, *p* = 0.011). In terms of comorbidities, the incidence of ischemic heart disease was significantly greater in the severe CKD (vs. control) (*p* = 0.016) and diabetes mellitus was likewise significantly greater in the severe CKD (vs. control) group (*p* < 0.001). There were no significant differences in depth of tumor invasion, lymph node metastasis or pathologic staging by group.

**Table 1:** Clinicopathologic variables in each CKD group.

	Control	Moderate CKD	P value^a^	Severe CKD	P value^b^
	60 ≤ eGFR	45 ≤ eGFR < 60		eGFR < 45	
	N = 637	N = 117		N = 44	
Age, years	65 (17–90)	75 (55–90)	<0.001	77.5 (38–87)	< 0.001
Gender [n (%)]			0.289		0.206
Male	403 (63.3)	80 (68.4)		32 (72.7)	
Female	234 (36.7)	37 (31.6)		12 (27.3)	
BMI, kg/m^2^	22.4 (14.9–44.0)	23.0 (15.7–31.5)	0.091	23.1 (16.4–32.1)	0.519
Albumin, g/dl	3.9 (2.0–4.7)	3.7 (2.5–4.6)	0.011	3.4 (1.9–4.4)	< 0.001
Ischemic heart disease [n (%)]			0.061		0.016
Present	32 (5.0)	11 (9.4)		6 (13.6)	
Absent	605 (95.0)	106 (90.6)		38 (86.4)	
Diabetes mellitus [n (%)]			0.094		<0.001
Present	69 (10.8)	19 (16.2)		13 (29.6)	
Absent	568 (89.2)	98 (83.8)		31 (70.4)	
Depth of tumor invasion (pT) [n (%)]			0.189		0.270
pT1, pT2	442 (69.4)	74 (63.3)		34 (77.3)	
pT3, pT4	195 (30.6)	43 (36.8)		10 (22.7)	
Lymph node metastasis (pN) [n (%)]			0.054		0.297
pN0	439 (68.9)	70 (59.8)		27 (61.4)	
pN1-pN3	198 (31.1)	47 (40.2)		17 (38.6)	
pStage [n (%)]			0.072		0.592
1	394 (61.9)	62 (53.0)		29 (65.9)	
2, 3	243 (38.2)	55 (47.0)		15 (34.1)	
Gastrectomy type [n (%)]			0.752		0.018
Total	139 (21.8)	24 (20.5)		3 (6.8)	
Partial	498 (78.2)	93 (79.5)		41 (93.2)	
Operative time, min	315 (118–963)	318 (152–730)	0.756	283 (188–656)	0.013
Blood loss, mL	50 (0–1530)	50 (0–4200)	0.936	68 (5–650)	0.288
Harvested lymph node count	32 (0–90)	31 (4–83)	0.068	28.5 (9–55)	0.070
Postoperative complications					
Leakage [n (%)]	21 (3.3)	6 (5.1)	0.327	6 (13.6)	<0.001
Pancreatic fistula [n (%)]	18 (2.8)	6 (5.1)	0.192	2 (4.6)	0.514
Intraabdominal abscess [n (%)]	22 (3.5)	3 (2.6)	0.621	0	0.210
Pneumonia [n (%)]	15 (2.4)	3 (2.6)	0.892	2 (4.6)	0.368
Major complication (CD≧3) [n (%)]	42 (6.6)	12 (10.3)	0.158	8 (18.2)	0.004
In hospital death [n (%)]	1 (0.2)	0	0.688	3 (6.8)	< 0.001
Postoperative stay, days	12 (6–105)	12 (6–72)	0.048	13 (9–291)	0.016
Postoperative chemotherapy	178 (27.9)	35 (29.9)	0.663	5 (11.4)	0.014

^a^ Control versus Moderate CKD,

^b^ Control versus Severe CKD.

*CKD* chronic kidney disease, *BMI*, body mass index; *CD*, Clavien Dindo classification.

### Operative findings and short-term outcomes

In type of gastrectomy, the proportion of partial gastrectomy was significantly greater in the severe CKD (vs. control) group (*p* = 0.018). Mean operative time was significantly shorter in the severe CKD (vs. control) group (*p* = 0.013). Mean blood loss estimates or mean counts of harvested lymph nodes did not differ significantly by group. The incidence of anastomotic leak was significantly greater in the severe CKD (vs. control) group (*p* < 0.001). The three groups exhibited similar rates of pancreatic fistula, intraabdominal abscess and pneumonia. Major complications (≥ CD 3) significantly occurred with greater frequency in the severe CKD (vs. control) group (*p* = 0.012). The incidence of in-hospital death was significantly greater in the severe CKD (vs. control) group (*p* < 0.001). Postoperative stays were significantly longer in the moderate and severe CKD (vs. control) group (*p* = 0.048, *p* = 0.016). Postoperative chemotherapy was performed significantly less frequently in patients with severe CKD (vs. control, *p* = 0.014).

### Correlation between anastomotic leak and clinical characteristics

The correlation between anastomotic leak and characteristic variables are shown in Table 2. Clinical characteristic variables that significantly correlated with the anastomotic leak were male (*p* < 0.001), CKD (*p* = 0.003), total gastrectomy (*p <* 0.001) and blood loss estimates (≥ 100 mL) (*p* = 0.019). Otherwise, age, BMI, albumin, diabetes mellitus, ischemic heart disease and pStage did not correlate with the incidence of anastomotic leak significantly. Uni and multivariate analysis of the risk factors for anastomotic leakage is shown in Table 3. In univariate analysis using age, gender, BMI, albumin, co-morbidities (ischemic heart disease, diabetes mellitus and CKD), gastrectomy type, pStage, blood loss and operative time as covariates, variables associated with anastomotic leak were male, severe CKD, total gastrectomy and blood loss. In multivariate analysis using gender, CKD, gastrectomy type and blood loss as covariates, male, severe CKD and total gastrectomy were all independently associated with the incidence of anastomotic leak as well. The hazard ratio for severe CKD was 4.63 (95% confidence interval [CI] 1.62–11.54; *p* = 0.006)

**Table 2:** Characteristic variables and anastomotic leakage.

		Anastomotic leak (+)	Anastomotic leak (-)	P value
		N = 33	N = 765	
Age, years	≥ 70	19 (57.6)	332 (43.4)	0.108
	< 70	14 (42.4)	433 (56.6)	
Gender [n (%)]	Male	30 (90.9)	485 (63.4)	< 0.001
	Female	3 (9.1)	280 (36.6)	
BMI, kg/m^2^	≥ 22	21 (63.6)	439 (57.5)	0.482
	< 22	12 (36.4)	325 (42.5)	
Albumin, g/dl	≥ 3.5	23 (69.7)	604 (79.0)	0.205
	< 3.5	10 (30.3)	161 (21.1)	
Diabetes mellitus [n (%)]	Absent	26 (78.8)	671 (87.7)	0.131
	Present	7 (21.2)	94 (12.3)	
Ischemic heart disease [n (%)] Absent		30 (90.9)	719 (94.0)	0.471
	Present	3 (9.1)	46 (6.0)	
CKD [n (%)]	Control	21 (63.6)	616 (80.5)	0.003
	Moderate	6 (18.2)	111 (14.5)	
	Severe	6 (18.2)	38 (5.0)	
Gastrectomy [n (%)]	Total	16 (48.5)	150 (19.6)	<0.001
	Partial	17 (51.5)	615 (80.4)	
pStage [n (%)]	1	16 (48.5)	469 (61.3)	0.140
	2,3	17 (51.5)	296 (38.7)	
Blood loss [n (%)]	≥ 100 mL	15 (45.5)	205 (26.8)	0.019
	< 100 mL	18 (54.6)	560 (73.2)	
Operative time [n (%)]	≥315 min	20 (60.6)	376 (49.2)	0.198
	< 315 min	13 (39.4)	389 (50.8)	

*BMI* body mass index, *CKD* chronic kidney disease.

**Table 3:** Uni- and multivariate analysis of the risk factors of anastomotic leakage.

		Univariate analysis		Multivariate analysis	
		HR (95% CI)	P value	HR (95% CI)	P value
Age, years	< 70	1			
	≥ 70	1.77 (0.88–3.65)	0.110		
Gender	Female	1		1	
	Male	5.77 (2.03–24.23)	< 0.001	4.47 (1.53–19.08)	0.004
BMI	< 22	1			
	≥ 22	1.30 (0.64–2.75)	0.479		
Albumin,g/dl	≥ 3.5	1			
	< 3.5	1.63 (0.73–3.41)	0.223		
Diabetes mellitus	Absent	1			
	Present	1.92 (0.75–4.33)	0.161		
Ischemic heart disease	Absent	1			
	Present	1.56 (0.37–4.60)	0.498		
CKD	Control	1		1	
	Moderate	1.59 (0.57–3.79)	0.351	1.56 (0.55–3.83)	0.377
	Severe	4.63 (1.62–11.55)	0.006	6.46 (2.13–17.74)	0.002
Gastrectomy	Partial	1		1	
	Total	3.86 (1.89–7.85)	< 0.001	3.95 (1.75–9.00)	0.001
pStage	1	1			
	2,3	1.68 (0.83–3.41)	0.145		
Blood loss	< 100mL	1		1	
	≥ 100mL	2.28 (1.11–4.60)	0.025	1.12 (0.50–2.44)	0.783
Operative time	< 315 min	1			
	≥ 315 min	1.56 (0.79–3.32)	0.196		

*HR* hazard ratio, *BMI* body mass index, *CKD* chronic kidney disease.

### Univariate and multivariate analyses of predicting factors of overall survival (OS) and Cancer-specific survival (CSS)

Univariate and multivariate analytic data for OS are shown in Table 4. Univariate analysis of OS indicated that age, gender, albumin, eGFR (severe CKD), total gastrectomy, depth of tumor, lymph node metastasis, complications (CD ≥ 3) and blood loss estimates (≥ 100 mL) were predictors of OS. In multivariate analysis, age (*p* < 0.001), eGFR (severe CKD) (*p* < 0.001), depth of tumor (*p* < 0.001), lymph node metastasis (*p* < 0.001) and complications (CD ≥ 3) (*p* = 0.002) were all independently associated with unfavorable outcomes for patients with GC. The hazard ratio (HR) for eGFR (severe CKD) was 3.11 (95% confidence interval [CI], 1.87–4.98). eGFR (severe CKD) was not a predictive factor for CSS by univariate analysis (univariate HR = 2.03; 95% CI = 0.85–4.09; *p* = 0.104); however, pT and pN stage were independent prognostic factors (Table 5).

**Table 4:** Uni- and multivariate analyses of factors predicting overall survival (OS).

	Univariate analysis	P value	Multivariate analysis	P value
	HR (95% CI)		HR (95% CI)	
Age, years (≥ 70)	2.05 (1.50–2.83)	< 0.001	1.83 (1.31–2.58)	< 0.001
Gender (Male)	1.69 (1.19–2.44)	0.003	1.36 (0.95–2.00)	0.095
Albumin, g/dl (< 3.5)	2.32 (1.67–3.19)	< 0.001	1.39 (0.97–1.98)	0.072
BMI, kg/m^2^ (< 22)	1.10 (0.81–1.51)	0.535	-	-
eGFR (Severe CKD)	3.68 (2.28–5.65)	< 0.001	3.11 (1.87–4.98)	< 0.001
Diabetes mellitus	1.54 (0.99–2.29)	0.053	1.09 (0.69–1.65)	0.702
Ischemic heart disease	1.53 (0.82–2.59)	0.169	-	-
Gastrectomy (Total)	1.74 (1.23–2.42)	0.002	1.06 (0.72–1.57)	0.780
Tumor depth (pT3,4)	3.64 (2.66–5.00)	<0.001	2.64 (1.82–3.86)	<0.001
Lymph node metastasis	3.93 (2.86–5.45)	<0.001	2.48 (1.73–3.58)	<0.001
Complication (CD ≥ 3)	2.83 (1.82–4.23)	<0.001	2.11 (1.33–3.22)	0.002
Blood loss, mL (≥ 100)	1.54 (1.11–2.11)	0.010	1.11 (0.78–1.60)	0.573

*HR* hazard ratio, *CI* confidence interval, *BMI* Body mass index, *CKD* Chronic renal disease, *CD* Clavien Dindo classification.

**Table 5:** Uni- and multivariate analyses of factors predicting Cancer-specific survival (CSS).

	Univariate analysis	P value	Multivariate analysis	P value
	HR (95% CI)		HR (95% CI)	
Age, years (≥ 70)	1.14 (0.74–1.76)	0.540	-	-
Gender, (Male)	1.08 (0.69–1.71)	0.751	-	-
Albumin, g/dl (< 3.5)	1.66 (1.02–2.62)	0.040	1.24 (0.75–1.98)	0.390
BMI, kg/m^2^ (< 22)	0.84 (0.53–1.29)	0.424	-	-
eGFR (Severe CKD)	2.03 (0.85–4.09)	0.104	-	-
Diabetes mellitus	1.36 (0.72–2.37)	0.326	-	-
Ischemic heart disease	1.78 (0.79–3.47)	0.150	-	-
Gastrectomy (Total)	3.09 (1.99–4.75)	< 0.001	1.21 (0.75–1.96)	0.430
Tumor depth (pT3, pT4)	15.8 (8.92–30.64)	< 0.001	7.84 (4.13–16.07)	< 0.001
Lymph node metastasis	9.29 (5.59–16.34)	< 0.001	3.48 (2.00–6.38)	< 0.001
Complication (CD ≥ 3)	1.96 (0.95–3.60)	0.068	1.15 (0.54–2.20)	0.692
Blood loss, mL (≥ 100)	2.38 (1.54–3.65)	< 0.001	1.14 (0.69–1.84)	0.608

*HR* hazard ratio, *CI* confidence interval, *BMI* Body mass index, *CKD* Chronic renal disease, *CD* Clavien Dindo classification.

### Long-term survival rate

**Fig 1:**
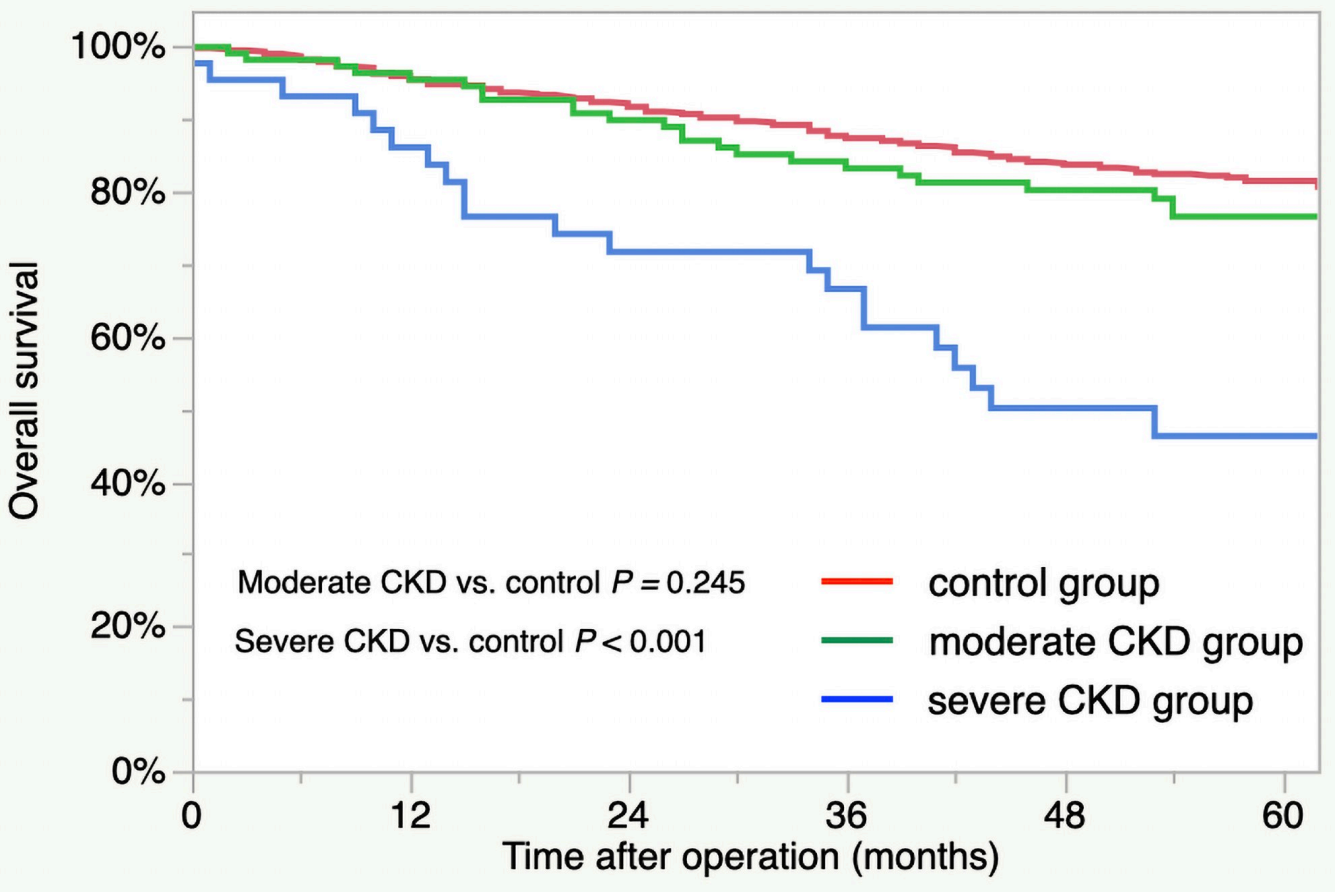
Overall survival of CKD groups: Control group (red line), moderate CKD group (green line), and severe CKD group (blue line).

**Fig 2:**
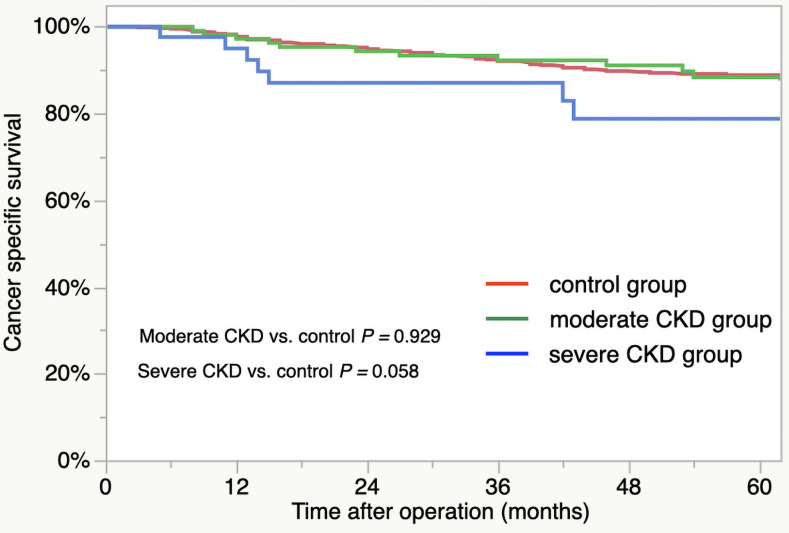
Cancer-specific survival of CKD groups: Control group (red line), moderate CKD group (green line), and severe CKD group (blue line).

For the patient survival curves, the 5-year OS rates for the groups were as follows: control group, 81.5%; moderate CKD group, 76.6%; severe CKD group, 46.3% (Fig 1). The OS outcomes were significantly worse for the severe CKD group compared to control group (severe CKD vs. control, *p* < 0.001). There was no significant difference in the OS between the control and moderate CKD groups (*p* = 0.245). The respective 5-year CSS rates of the three group were as follows: control, 88.9%; moderate CKD, 88.3%; severe CKD, 78.8% (Fig 2). The outcomes did not differ significantly by group (moderate CKD vs. control, *p* = 0.929, severe CKD vs. control, *p* = 0.058).

## Discussion

In this study, CKD was closely associated with short-term outcomes in GC patients who underwent laparoscopic gastrectomy. Severe CKD (eGFR < 45) was a risk factor for major complications, in-hospital death, and longer hospital stays. In particular, severe CKD (eGFR < 45) emerged as an independent predictor of anastomotic leak. For long-term outcomes, the OS of patients was significantly worse for the severe CKD group than the other two groups; however, the CSS rate did not differ between groups.

Our results indicate that CKD adversely affects the short- and long-term outcomes of GC patients undergoing laparoscopic gastrectomy. Matsumoto et al. reported the outcomes of gastrectomy for GC patients with CKD, in which about 85% of the patients underwent open gastrectomy [12]. In their study, the percentages of major complications (CD ≥ 3) for the different CKD groups were 34.4% (eGFR ≥ 60), 47.3% (30 < eGFR ≤ 60), and 61.2% (eGFR < 30). The incidence of complications was much greater in every CKD grade compared to those in our study. This discrepancy may be the result of the different approaches (laparoscopic vs. open). Because laparoscopic surgery results in less blood loss than open surgery [3, 21], there would be less change in the intraoperative water balance, which may be beneficial for patients with CKD as it would reduce postoperative tissue edema and maintain peripheral tissue blood flow, reducing complications. However, Wakahara et al. [14] reported that GC patients with CKD have similar complication rates and relapse-free survival rates to GC patients without CKD, indicating that CKD did not affect the short-and long-term outcomes of these patients. Thus, a multi-institutional study is needed to fully elucidate the association between GC patients with CKD and treatment outcomes.

In this study, even with minimally invasive surgery, the incidence of anastomotic leak increased with worsening CKD grade. This incidence rate in the severe CKD group (eGFR < 45) was more than fourfold higher than in the control group. In patients with gastrointestinal cancer, impaired renal function can increase the incidence of anastomotic leak [12, 13]. However, the optimal cutoff value of eGFR for predicting anastomotic leak in patients undergoing laparoscopic gastrectomy is unclear. Tanaka et al. showed that eGFR was an independent risk factor for leakage in patients with clinical T2-4 GC undergoing gastrectomy with D2 lymphadenectomy [13]. Their optimal cutoff for eGFR in predicting anastomotic leak was 63.2, which is far higher than our cutoff value (eGFR 45). One possible explanation is that patients with stage 1 GC disease were excluded in their study, whereas our study was comprised of patients with disease stages 1–3. Another reason may be that most patients enrolled in their study might have undergone open surgery because of advanced GC, although the surgical approach was not described. Different patient characteristics (e.g., surgical approach and TNM stage) may have also affected the incidence of anastomotic leak. Although there are a few reports that eGFR affects treatment outcome [12, 13], an optimal cut-off value for predicting outcome based on CKD grade has not been discussed. In addition, they include a lot of open gastrectomy. Our results showed that severe CKD (eGFR < 45) significantly increases the rate of anastomotic leak and significantly decreases the overall survival rate. Therefore, we emphasize that GC patients with an eGFR value less than 45 (CKD > G3b) should be treated for CKD both preoperatively and postoperatively.

In this study, 5-year OS differed significantly by CKD group, regardless of their similar rates of TNM stage. The control and moderate CKD groups had similar 5-year OS rates, but the patient outcomes for severe CKD were significantly worse than those of the control. One of our presumptions is that patients in the severe CKD group may not have received sufficient postoperative chemotherapy. Many CKD patients (severe CKD; eGFR < 45) who undergo gastrectomy cannot tolerate continuous chemotherapy. Another reason is that patients with severe CKD (eGFR < 45) had a higher incidence of anastomotic leak than the other two groups. Because postoperative infectious complications have been reported to lead to a poor prognosis [22], the high complication rate in patients with severe CKD may result in a worse survival rate. Furthermore, Foley et al. maintain that patients with CKD are plagued by catabolic conditions that lead to muscle atrophy, with eGFR and sarcopenia showing a single correlation [23]. In our view, many patients with severe CKD (eGFR < 45) have sarcopenia preoperatively, and decreased dietary intake after gastrectomy further worsens their nutritional status, leading to an increase in deaths from infectious diseases. More work is needed to confirm these hypotheses.

Unfortunately, the ability to improve eGFR before surgery remains challenging. However, there are several approaches that can improve CKD according to clinical practice guidelines, including stopping smoking and excessive drinking and maintaining a proper weight [11, 24]. For diet therapy, salt intake should be limited to 3–6 g/day and protein intake to 0.6–0.8 g/kg/day for CKD classifications greater than G3a. For CKD with hypertension, blood pressure should be maintained at 130–140/80–90 mmHg using antihypertensive drugs. When hemoglobin levels are less than 11 g/dL, administration of an erythropoietin stimulating antagonist is recommended. We believe that these clinical approaches should be reinforced before surgery for severe CKD in GC patients.

As already mentioned, it is common to find that patients with CKD are in catabolic states due to suppression of protein synthesis, and are subject to muscle wasting [9]. Because declines in muscle mass have been shown to negatively impact outcomes of patient with GCs [25, 26], blockade of CKD-induced protein loss may offer a new therapeutic strategy to improve long-term results. In a previous report by Wang et al [9], the expression of myostatin, a negative regulator of skeletal muscle growth, appears to increase in patients with CKD. Inhibition of myostatin is known to increase in IGF-1/insulin/PI3K/Akt intracellular signaling, providing a mechanism for muscle growth. Another benefit of myostatin inhibition is a cited decrease in circulating levels of TNF-α, IL-6, INF-γ and macropharge colony-stimulating factor 1, all of which are associated with loss of muscle mass [27]. Such an approach may be particularly useful for improving outcomes of gastrectomy in patients with GC and CKD.

The present investigation has several limitations. First, this study was a retrospective study based on a limited number of patients. Thus, our findings are not definitive. A multicenter study is assuredly needed for validation. Another issue is that only eGFR was used to gauge renal function. It is essential to include creatinine clearance (CCr) to evaluate exact renal function; however, no CCr data was available. Therefore, the results of this study need to be confirmed by incorporating CCr. Third, postoperative chemotherapy, including regimen and dose, was not considered in the present study. The CKD patients with advanced-stage GC disease may have received chemotherapy at less than the standard dose. Finally, the concept that renal function at one preoperative time point would impact long-term patient outcomes has not been formally corroborated.

## Conclusions

Our results indicate that eGFR is a useful tool for predicting the risk of anastomotic leak and OS for GC patients undergoing laparoscopic gastrectomy. This parameter is informative for understanding the surgical risk and long-term outcomes of GC patients. Clinical care to improve eGFR should be reinforced at the time of GC treatment for GC patients with severe CKD.

## Supporting information

S1 Table (XLSX)
